# DU‐Vigilance; Capturing Unsuspected Problems in Drug Utilization

**DOI:** 10.1002/pds.70423

**Published:** 2026-07-30

**Authors:** Jesper Hallas, Hanin Harbi, Thomas Delvin, Lotte Rasmussen

**Affiliations:** ^1^ Clinical Pharmacology, Pharmacy and Environmental Medicine University of Southern Denmark Odense Denmark

**Keywords:** aberrant prescribing, drug surveillance, drug utilization, pharmacovigilance, prescription databases, real‐world data

## Abstract

- Pharmacovigilance should expand beyond adverse drug reactions.
- A concept, Drug Utilization Vigilance (DU‐vigilance), is proposed as an approach to detect unsuspected prescribing or utilization problems.
- DU‐vigilance relies on analyses of large‐scale real‐world data.
- Aberrant drug utilization is dynamic and requires continuous surveillance.
- Integrating DU‐vigilance within traditional pharmacovigilance could improve healthcare quality.

Pharmacovigilance is defined as “the science and activities relating to the detection, assessment, understanding and prevention of adverse effects or any other medicine‐related problem” [1]. While most pharmacovigilance activities have focused on the first part of this definition—adverse effects—the second part, “any other medicine‐related problem,” has been relatively neglected. We suggest that this imbalance can be addressed through the concept of Drug Utilization Vigilance (DU‐vigilance), which applies the principles of pharmacovigilance to the detection and prevention of anomalous utilization patterns. The novelty of this proposal lies in its systematic, hypothesis‐generating application aimed at identifying anomalous utilization patterns that were not suspected a priori, rather than in surveys of known or anticipated problems.

The ultimate objective of drug utilization research is to facilitate the rational use of drugs in populations [2]. It has a long tradition, particularly in evaluating new medicines, drugs of obvious public health interest, or where problems are suspected in advance. Yet this practice is usually rooted in established treatment guidelines, rarely addressing utilization problems that are unsuspected. In this respect, DU‐vigilance is conceptually closer to signal detection in pharmacovigilance than to descriptive or guideline‐driven drug utilization surveys.

Unlike traditional pharmacovigilance, DU‐vigilance cannot rely on spontaneous reports since the problems would rarely be visible to the individual health care professional. Instead, it must use large‐scale real‐world data sources such as prescription registries, electronic health records, and insurance claims. These allow systematic, high‐throughput screening for unsuspected anomalous utilization. The analytical tools underlying DU‐vigilance are not novel in themselves and have long been part of the Drug Utilization Research toolbox, with approaches for identifying, for example, unexpected use of high doses or low doses, unusually long or short treatment durations, and relapse pattern (Table 1). The finding of anomalies is not necessarily based on guidelines but—at least in our experience—arise more often from review of findings by a subject matter expert. Proof‐of‐concept studies have demonstrated that hypothesis‐generating screening of prescription data can reveal such anomalies, even when they are not suspected beforehand [7, 10].

**TABLE 1:** Tools for capturing unsuspected problems in drug utilization, that is, DU‐vigilance, (upper part) and tools dedicated to specific drug utilization problem (lower part).

Type	Tool	Examples of problems captured
Simple quantitative measures, variability over time or region	Therapeutic intensity (DDD/inh/day) [3] Number of users Drug expenditure per capita Rate of new users Prevalence of users DU90 [4]	Unexpected regional variability, overutilization of second‐line or expensive drugs, dramatic temporal changes, adherence to formulary
Advanced general methods	Lorenz curve [5]	Abuse potential, heavy users, frequent sporadic users
	Prevalence: incidence ratio [5]	Short‐ or long term use
	Drug survival [6]	Short survival of drug treatment implying adverse effects or lack of efficacy. Long survival implying off‐label use or rebound effects
	Waiting time distribution [7, 8]	Undue short‐ or long term use, seasonality, relapse liability, sporadic use
	Doctor shopping [9]	Abuse potential
Tools dedicated to specific drug utilization problem	Any analysis adapted to a pre‐specified problem. Undue variability over time or region may imply unsuspected prescribing problem	For example, underuse of inhaled corticosteroids in asthma patients, overuse of broad spectrum antibiotics for specific infections, etc.

A key difference between ADRs and anomalous utilization is their stability. ADRs are generally stable once identified. A drug that is hepatotoxic today will remain so tomorrow. Aberrant utilization, by contrast, is dynamic. It can emerge rapidly through practice drift, off‐label adoption, or changes in reimbursement and guidelines. In fact, a dramatic temporal or regional shift in utilization parameters may be exactly what triggers suspicions of anomalous utilization that may be worth pursuing by dedicated tools (Table 1). This means DU‐vigilance requires continuous monitoring of drug utilization patterns, making it a natural complement to traditional ADR surveillance.

Drug utilization studies address problems that matter greatly to regulators, payers, and healthcare systems. While ADR monitoring often concerns rare events, anomalous utilization may be common, costly, and immediately impactful. By recognizing anomalous utilization as a pharmacovigilance concern, we can ensure that these issues receive systematic attention and contribute directly to safer and more sustainable prescribing. DU‐vigilance would most naturally be embedded within existing regulatory pharmacovigilance structures and conducted by public authorities with access to large‐scale utilization data and relevant analytical expertise. As with adverse drug reaction surveillance, findings should be considered hypothesis‐generating and subject to further evaluation before any regulatory or policy action should be contemplated.

By incorporating systematic monitoring of prescribing patterns into existing frameworks, pharmacovigilance can become more comprehensive, providing due attention to “any other medicine‐related problem.”

## Funding

The authors have nothing to report.

## Conflicts of Interest

The authors declare no conflicts of interest.
